# Whole grain products in Brazil: the need for regulation to ensure nutritional benefits and prevent the misuse of marketing strategies

**DOI:** 10.11606/s1518-8787.2023057004790

**Published:** 2023-09-14

**Authors:** Giovanna Calixto Andrade, Laís Amaral Mais, Camila Zancheta Ricardo, Ana Clara Duran, Ana Paula Bortoletto Martins

**Affiliations:** I Universidade de São Paulo Faculdade de Medicina Departamento de Medicina Preventiva São Paulo SP Brasil Universidade de São Paulo. Faculdade de Medicina. Departamento de Medicina Preventiva. São Paulo, SP, Brasil; II Universidade de São Paulo Núcleo de Pesquisas Epidemiológicas em Nutrição e Saúde São Paulo SP Brasil Universidade de São Paulo. Núcleo de Pesquisas Epidemiológicas em Nutrição e Saúde. São Paulo, SP. Brasil; III Instituto Brasileiro de Defesa do Consumidor São Paulo SP Brasil Instituto Brasileiro de Defesa do Consumidor. São Paulo, SP, Brasil; IV Universidad de Chile Escuela de Salud Pública Facultad de Medicina Santiago Chile Universidad de Chile, Escuela de Salud Pública, Facultad de Medicina. Santiago, Chile; V Universidade Estadual de Campinas Núcleo de Estudos e Pesquisas em Alimentação Campinas SP Brasil Universidade Estadual de Campinas. Núcleo de Estudos e Pesquisas em Alimentação. Campinas, SP, Brasil

**Keywords:** Whole Grains, Food Labeling, Nutritive Value

## Abstract

**OBJECTIVE:**

This study aims to evaluate the use of “whole grains” claims in food products marketed in Brazil and evaluate the nutrient profile of these products.

**METHODS:**

Data from 775 grain-based packaged foods collected in Brazil from April to July 2017 were analyzed. Based on the INFORMAS protocol for food labeling, the prevalence of packaged foods with “whole grains” claims was estimated. Information on the list of ingredients was analyzed to evaluate the presence and amount of whole or refined grains in six food groups. The nutrient profiles of the products with and without “whole grains” claims were compared using the Pan American Health Organization (PAHO) nutrient profile model.

**RESULTS:**

The packages of about 19% of the evaluated products showed “whole grains” claims in their front panel. Of these, 35% lacked any whole grains among their top three ingredients. Breakfast cereals, granola bars, bread, cakes and other bakery products, cookies, and pasta had higher amounts of refined flour than whole grain ingredients in their compositions.We found 66% of products with “whole grains” claims were high in nutrients of concern according to PAHO’s criteria.

**CONCLUSION:**

Our results showed that over a third of the products in Brazil with “whole grains” claims lacked whole grains as one of their main ingredients. Most had a high content of nutrients associated with noncommunicable chronic disease risk factors, indicating the overestimation of their health benefits.

## INTRODUCTION

Whole grain is defined as the product from cereal processing that maintains its fractions that characterize the whole grain, such as the endosperm, germ, and bran. Whole grain consumption has been associated with nutrient intake (such as dietary fibers, complex B vitamins, vitamin E, selenium, zinc, copper, and magnesium) and good diet quality^1^. Additionally, studies have indicated a positive association between the consumption of whole grains and a reduced risk of type 2 diabetes, obesity, coronary heart disease, cardiovascular disease, total cancer, and mortality from all causes^2^.

Despite these benefits, a national study in Brazil, conducted with a representative sample of the population in 2008/2009, showed that the consumption of whole grains was much lower than that of refined grain products. For example, the mean daily consumption of white rice totaled 160.3 g/day; whereas the mean daily consumption of brown rice, only 8.1 g/day; the average consumption of white bread, 53.0 g/day; whereas that of whole grain bread, 0.9 g/day^5^.

The World Health Organization (WHO) recommends that countries develop and implement strategies to promote the consumption of whole grains in food and nutrition public policies^6^. However, up to 2021, the Brazilian legislation required neither clear information nor minimum amounts of whole grains in food and beverage packages.

The Resolution of the National Commission on Standards and Food Standards (*Comissão Nacional de Normas e Padrões para Alimentos* – CNNPA) n^o^ 12, published in 1978, included the definition of whole grain products^7^. This resolution was replaced in 2005 by the Resolution of the Collegiate Board (*Resolução da Diretoria Colegiada* – RDC) n^o^ 263/2005^8^. In the new regulation, the National Health Regulatory Agency (*Agência Nacional de Vigilância Sanitária* – Anvisa) neither defined whole grain products nor foresaw the minimum amount of wholemeal flour necessary for claiming “whole grain” use in food products sold in Brazil. Companies are not required to declare the percentage of wholemeal used on food labels^8^.

Considering the absence of a definition, composition criteria, and labeling standards for whole grain-based food products, Anvisa reviewed the regulation of these products. The agency recognized that the absence of a whole grain definition, the high proportions of refined ingredients in the formulation of whole grain-based foods, and the lack of surveillance on the use of “whole grains” claims in food package constituted the main causes of information asymmetry, which can lead to consequences such as inadequate food choices by consumers^9^.

After conducting a regulatory process to improve these legislation gaps, Anvisa published a new regulation for grain-based food products in 2021, which will be implemented from 2023 onward. To claim “whole grain” use, products must have at least 30% of whole grains. Additionally, the amount of whole grains should exceed that of refined grains (otherwise, the claim must also have the percentage of whole grains in the composition of the product and its list of ingredients)^10^.

Considering the health benefits associated with the consumption of whole grains and the potential misunderstanding of the use of “whole grains” claims in cereal-based products, this study assessed the prevalence of packaged foods sold in Brazil with claims of “whole grains” and their nutrient profile before the implementation of the new national regulation.

## METHODS

This is a cross-sectional study that used information collected from labels of packaged foods and beverages sold in the five food retail chains with the largest market shares in Brazil from April to July 2017.

Information from Euromonitor International annual sales was used to select the supermarket chains from which data was to be collected^11^. The addresses of the selected supermarket chain stores were geocoded and classified according to neighborhood income tertiles. To estimate the neighborhood income level of each supermarket, a buffer of one km around each store was measured. Then, information on the mean income of the main house breadwinners, available in the Brazilian Demographic Census 2010^12^, was used. In total, two stores of each food retail chain were chosen (one from the first tertile and one from the last tertile), prioritizing those with the largest physical areas. All supermarket chains included in this study provided formal permission for data collection.

All package panels of all foods and beverages found in each food store were photographed by trained fieldworkers, following the methods in Kanter et al.^13^ Then, the available information on the labels, such as the description of the product, brand, origin, list of ingredients, and information from the nutrition facts panel, were entered by trained typists in the online platform RedCap using a form developed by the University of North Carolina at Chapel Hill (UNC) in the United States (US) and by the *Instituto de Nutrición y Tecnología de los Alimentos* (INTA), in Chile, adapted to Brazil. Duplicated items and products lacking a nutrition facts panel, available in more than one package size, and with multiple items were excluded. Information from 11,434 products was collected.

A sample of 30% of all products (3,491 products) was randomly selected to estimate the prevalence of claims and other marketing strategies in food packages. Only grain-based products were included in this study, totaling 775 products. The selected food items were sorted into seven groups: bread; cakes and other bakery products, breakfast cereals and granola bars, cereal grain and flours, cookies, pasta, and savory snacks.

The International Network for Food and Obesity/Non-Communicable Diseases (NCDs) Research, Monitoring, and Action Support (INFORMAS) was used to classify health and nutrition claims according to format, type, class, and content. The INFORMAS protocol stands out for being an internationally standardized methodology, enabling the comparison of studies evaluating the marketing of packaged foods and beverages over time and across countries^14^.

Among the products with “whole grains” claims, the number of times a claim appeared on a package (whether it was verbal, numerical, or symbolic) and the prevalence of products that claimed percentages of whole grains in their list of ingredients were assessed. Intra- and interrater reliability analyses were performed^15^.

Lists of ingredients were used to verify whether whole grains were the main ingredients in the assessed products. By the Brazilian regulation, foods and beverages are required to list their ingredients in descending order^16^. Because of that, the top three ingredients in lists were examined. First, the prevalence of products with “whole grains” claims that had whole grains as their first ingredient and among the top three ingredients were estimated. Then, the most frequent items in the first three positions in ingredient lists were computed. As we aimed to evaluate the degree of refinement rather than the type of cereal, different cereals (such as corn, rice, oats etc.) were combined according to the industrial processing level in “whole grain” or “refined grain.”

Finally, the proportion of grain-based food products high in nutrients of concern (total fat, saturated fat, trans fat, free sugar, and sodium) and/or with any amount of noncaloric sweeteners according to the Pan American Health Organization (PAHO) nutrient profile model were estimated. The criteria to define the PAHO nutrient profile model were based on WHO recommendations focusing on the prevention of obesity and non-communicable diseases (NCDs). PAHO criteria also consider the purpose and extent of industrial processing foods underwent, excluding unprocessed and minimally processed foods from its classification^17^. More details about the PAHO nutrient profile model can be accessed in the Supplementary Material 1^a^.

The nutrient content of food products was evaluated using the information available on the mandatory nutrition fact panels on the label of packaged foods sold in Brazil^18^. Although the PAHO nutrient profile model considers free sugars, this information is optionally available on the labels of food products sold in Brazil. When available, added sugar content was used to estimate free sugars amounts, following the PAHO method, which considers information on the amount of sugars on food labels^17^. However, analyses that considered total or free sugars were conducted for the sub-sample of products (12.4%) that provided such information. Thus, the prevalence of products with high sugar content is underestimated.

Confidence intervals (95% CI) were used to compare the prevalence of products with a high content of nutrients of concern in foods with and without “whole grains” claims.

## RESULTS

We evaluated 775 packages of grain-based food products, of which 147 (19.0%) contained at least one claim of “whole grains.” This claim occurred more often in breads (31.8%), followed by cereal grains and flours (31.3%), breakfast cereals and granola bars (21.0%), cookies (19.6%), pastas (13.2%), cakes and other bakery products (7.4%), and savory snacks (3.7%) (Table 1).

**Table 1:** Prevalence of “whole grains” claims on the packages of grain-based food products marketed in Brazilian supermarkets, 2017 (n = 775).

Food categories	n	Products with “whole grains” claims

		% (95%CI)
Breads	107	31,8 (23,5;41,3)
Cakes and other bakery products	68	7,4 (3,0;16,8)
Breakfast cereals and granola bars	95	21,0 (14,0;30,5)
Cereal grains and flours	96	31,3 (22,7;41,2)
Cookies	225	19,6 (14,9;25,3)
Pastas	76	13,2 (7,1;22,8)
Savory snacks	108	3,7 (1,4;2,5)
Total	775	19,0 (16,3;21,9)

95%CI: 95% confidence interval.

In many products, “whole grains” claims appeared repeatedly throughout packages. Savory snacks, breakfast cereals, and granola bars had a “whole grains” claim up to three times in their packages; in cakes and other bakery products, up to five times; in breads, cookies, and pastas packages, up to eight times; and in cereal grains and flours up to 15 times (data not shown). In all food groups, we found more frequent verbal claims (90.5%), followed by numerical (21.8%) and symbolic (2.0%) claims (Table 2). ‘Whole grain,’ ‘made with wholemeal,’ ‘100% whole grain,’ and ‘5 whole grains’ were the most frequently found verbal and numerical claims, and an ear of wheat was the most used image. Only 15.0% of breakfast cereals and granola bars, 9.1% of cookies, 5.0% of pastas, and 3.3% of cereal grains and flours packages voluntarily reported the percentage of whole grains in their ingredient lists. No assessed breads, cakes and other bakery products, and snacks had such information (data not shown).

**Table 2:** Percentage of different types of “whole grains” claims found in the food packages of grain-based food products with “whole grains” claim marketed in Brazil, 2017 (n =147).

Food categories	n	Products with “whole grains” claim		

		Numeric (%)	Verbal (%)	Symbolic (%)
Breads	34	17.7	94.1	2.9
Cakes and other bakery products	5	80.0	100	0.0
Breakfast cereals and granola bars	20	30.0	90.0	5.0
Cereal grains and flours	30	23.3	86.7	3.3
Cookies	44	20.4	86.4	0.0
Pastas	10	0.0	100.0	0.0
Savory snacks	4	0.0	100.0	0.0
**Total**	**147**	**21.8**	**90.5**	**2.0**

Table 3 describes the most frequent ingredients in the composition of grain-based food products. Among products with “whole grains” claims, only 49.7% had a whole grain as their first ingredient, and 65.3% showed whole grains among the first three ingredients. Breakfast cereals, granola bars, and pastas were the foods with the lowest percentage of products with whole grains among the top three ingredients. Moreover, we found sugar among the main ingredients of breakfast cereals and granola bars, breads, cakes, other bakery products, and cookies. Vegetable fat was the second ingredient most frequently found in savory snacks.

**Table 3:** Percentage of products with whole grains among the top three ingredients in the list of ingredients and the main ingredients of grain-based food products with “whole grains” claims on their labels, 2017 (n = 147).

Food categories	Products in which whole grains comprise most of their composition (%)	Products with whole grains among the top three ingredients	Ingredients that contribute the largest amount in products presenting “whole grains” claims		
	
		(%)	1^st^	2^nd^	3^rd^
Breads	58.8	88.2	Whole grain	Refined grain	Sugar
Cakes and other bakery products	80.0	80.0	Whole grain	Egg	Sugar
Breakfast cereals and granola bars	25.0	30.0	Refined grain	Sugar	Whole grain
Cereal grains and flours	76.7	76.7	Whole grain	Refined grain	*
Cookies	40.9	61.4	Refined grain	Whole grain	Sugar
Pastas	10.0	30.0	Refined grain	Eggs	Whole grain
Savory snacks	50.0	75.0	Whole grain	Vegetable fat	Refined grain
Total	49.7	65.3	-	-	-

*Most products in the ‘Cereal grains and flours’ group contained only one or two ingredients in their list of ingredients. The products with three or more ingredients greatly varied regarding *in natura* and minimally processed food items, making it impossible to identify a more frequent ingredient.

About 90.0% of breakfast cereals and granola bars, breads, savory snacks, and cookies showed considerable amounts of at least one of the assessed nutrients of concern (total fat, saturated fat, trans fat, free sugar, and sodium) according to the PAHO criteria. We found a similar prevalence of foods with at least one warning of high content of nutrients of concern among products with and without “whole grains” claims in the package (Table 4).

**Table 4:** Prevalence of grain-based food products with a high content of at least one nutrient of concern (total fat, saturated fat, trans fat, free sugar, sodium, and sweetener) according to the Pan American Health Organization (PAHO) nutrient profile model, 2017 (n = 775).

Food categories	Presence of “whole grains” claims	

	No	Yes
	
	(n = 628)	(n = 147)
	
	% (95%CI)	% (95%CI)
Breads	95,9 (87,7;98,7)	88,2 (71,4;95,7)
Cakes and other bakery products	85,7 (74,4;92,5)	100
Breakfast cereals and granola bars	62,7 (51,0;73,0)	70,0 (45,0;86,9)
Cereal grains and flours	1,5 (0;10,5)	0,0
Cookies	93,9 (89,3;96,6)	93,2 (80,1;97,9)
Pastas	30,3 (20,2;42,7)	30,0 (7,6;69,0)
Savory snacks	93,3 (86,4;97,0)	100,0
**Total**	**73,1 (69,5;76,4)**	**66,0 (57,9;73,3)**

95%CI: 95% confidence interval.

## DISCUSSION

Using “whole grains” claims is common in the grain-based food products marketed in Brazil. Although many products use the “whole grains” claim in their package, only a few show percentages of whole grains and not all contain large amounts of wholemeal flour. Additionally, some of these products have high contents of nutrients of concern, such as total fat, saturated fat, trans fat, free sugars, and sodium or even the presence of noncaloric sweeteners^19^.

We found that only half of the products with a “whole grains” claim indeed had whole grains as the most abundant component in their list of ingredients, which can mislead consumers seeking whole- grain foods. Moreover, many of these products had substantial amounts of refined wheat and other cereal-based flours, added sugars, and saturated and trans fats, which are associated with an increased risk of NCDs^17,19^.

We found a similar prevalence of products with at least one warning of high content of critical nutrients according to PAHO criteria among products with and without “whole grains” claims in their packages. Breads, cakes, other bakery products, savory snacks, and cookies were the food categories with the highest number of products with an excess of nutrients of concern. Cereals, flours, and pastas were the food categories with the lowest proportion of products with high critical nutrient content. Unsurprisingly, these categories consist of unprocessed and minimally processed foods, which have better nutrient profiles than ultra-processed food products^17,23^.

Previous studies have found equivalent results. In Brazil (2014), a study evaluating the composition of breads with “whole grain” claims showed that 60% of the assessed brands lacked wholemeal flour as their first ingredient^24^. In Canada (2013), a study evaluating breads with “whole grains” claims showed that only 54% of products showed a whole grain cereal as the first component in their ingredient lists. Additionally, products with “whole grains” claims were significantly higher in energy, total fat, and sugars than those without these claims^25^. Breakfast cereals marketed to children in the US (2008) with a “whole grains” claim had a higher content of fat and a lower content of sugar than products without the claim^26^.

The low amount of wholemeal flour in products with “whole grains” claims stems from gaps in Brazilian food regulations. The lack of definition of what a whole grain is and the absence of rules for the use of “whole grains” claims enabled the food industry to include refined grains in products that carry misleading claims for whole grains. Some of the evaluated products, for example, combined refined flour with wheat bran as “reconstituted wholemeal flour.” This combination contains no wheat germs and is thus not considered wholemeal. Other products declared the presence of grains in their list of ingredients but without defining their refinement degree.

New regulations for the use of “whole grains” claims in foods sold in Brazil were approved in 2021 and will go into effect in late 2023. A clearer definition of whole grain flour was approved. Food labels will also have to offer information on the proportion of whole grains in their products^10^. Despite improvements, the minimum amount of whole wheat flour for a product to receive a “whole grains” claim is lower than the cutoffs approved in other countries. In the US, breads and pastas can only carry a “whole grains” claim if they have no refined flour. The use of “whole grains” claims defines no minimum amount of wholemeal flour in products^27^. Similarly, in the United Kingdom (UK), breads can receive a “whole grains” claim if they only have wholemeal flour^28^. These examples show that the food industry can offer products with larger proportions of wholemeal flour than what we found in Brazil.

In addition to improvements on the information related to the content of whole grains in food labels, health authorities should consider banning claims on the package of foods with a high amount of nutrients of concern, which would prohibit products with poor nutrient profiles from being marketed with the “whole grains” claim and help to prevent consumers from being deluded.

This is the first study that evaluates the use of “whole grains” claims and the nutrition profile of packaged grain-based food products marketed in Brazil. Additionally, this study stands out for its sample size and use of the PAHO nutrient profile model, specifically developed for Latin America based on scientific evidence by an Expert Consultation Group. Nonetheless, we could neither perform laboratory analysis to verify the number of whole grains in the assessed products nor evaluate their nutrient content. Thus, we relied on the information available on the food labels. However, the nutrition information on the labels of food products marketed in Brazil has been used without any indication that the percentage of erroneous information is relevant^29^. The lack of information on free sugar content on the label of products sold in Brazil probably caused a lower prevalence of products with a high content of critical nutrients but we believe that this information is underestimated for products with and without “whole grains” claims.

We should mention that we conducted our analyses before the implementation of the new regulation for foods with whole grains. These findings will enable us to evaluate whether the new labeling regulations for whole grains will be fully implemented in the coming years and to what extent they will lead to changes in the nutrient profile of food products. It will also enable us to evaluate the content of whole grains in foods sold in Brazil by comparing the information we gathered on food labels with similar information to be collected after the new regulation implementation. Thus, we anticipate that several cereal-based products will undergo reformulation to maintain their “whole grains” claims.

## CONCLUSION

A considerable proportion of products with a “whole grains” claim in Brazil show no considerable amounts of wholemeal grains and may have a high content of nutrients associated with increased food-related NCD risks. When implemented, the new Brazilian labeling regulations for whole grains will demand that food companies include information on the proportion of whole grains on food labels. The results in this study will serve as a baseline for assessing the impact of new labeling regulations for the use of “whole grains” claims, enabling comparisons of these products before and after the new regulations.

## Footnotes

a Available from: https://www.fsp.usp.br/nupens/wp-content/uploads/2023/09/Supplementary-Material_LAM_REVIEWCLEAN.docx
